# Supplementary material for: A large-scale genome-wide association and meta-analysis identified four novel susceptibility loci for leprosy
Source: Nat Commun. 2016 Dec 15;7:13760. doi: 10.1038/ncomms13760 (PMC5172377; doi:10.1038/ncomms13760)
Supplement: Supplementary Information — Supplementary Figures 1-3 and Supplementary Tables 1-6 [file ncomms13760-s1.pdf]

Supplementary Figures

Supplementary Figure 1 Principal components analysis (PCA) of all samples analyzed in the discovery phase. Colors represent the phenotype of study populations. a) The first sample (GWAS1) was the previously published GWAS dataset of leprosy, consisting of 706 leprosy cases, 1,223 healthy controls, all of northern Chinese Han decent; b-d) The second one (GWAS2) was a newly published dataset of 840 leprosy cases and 924 controls from northern (Chinese Han, **b**) and southern China (Chinese Han **c** and ethnic minorities, **d**); e-f) The third sample was a new GWAS dataset (GWAS3) of 1197 leprosy cases and 1426 controls from northern (Chinese Han, **e**) and southern China (Chinese Han, **f**)

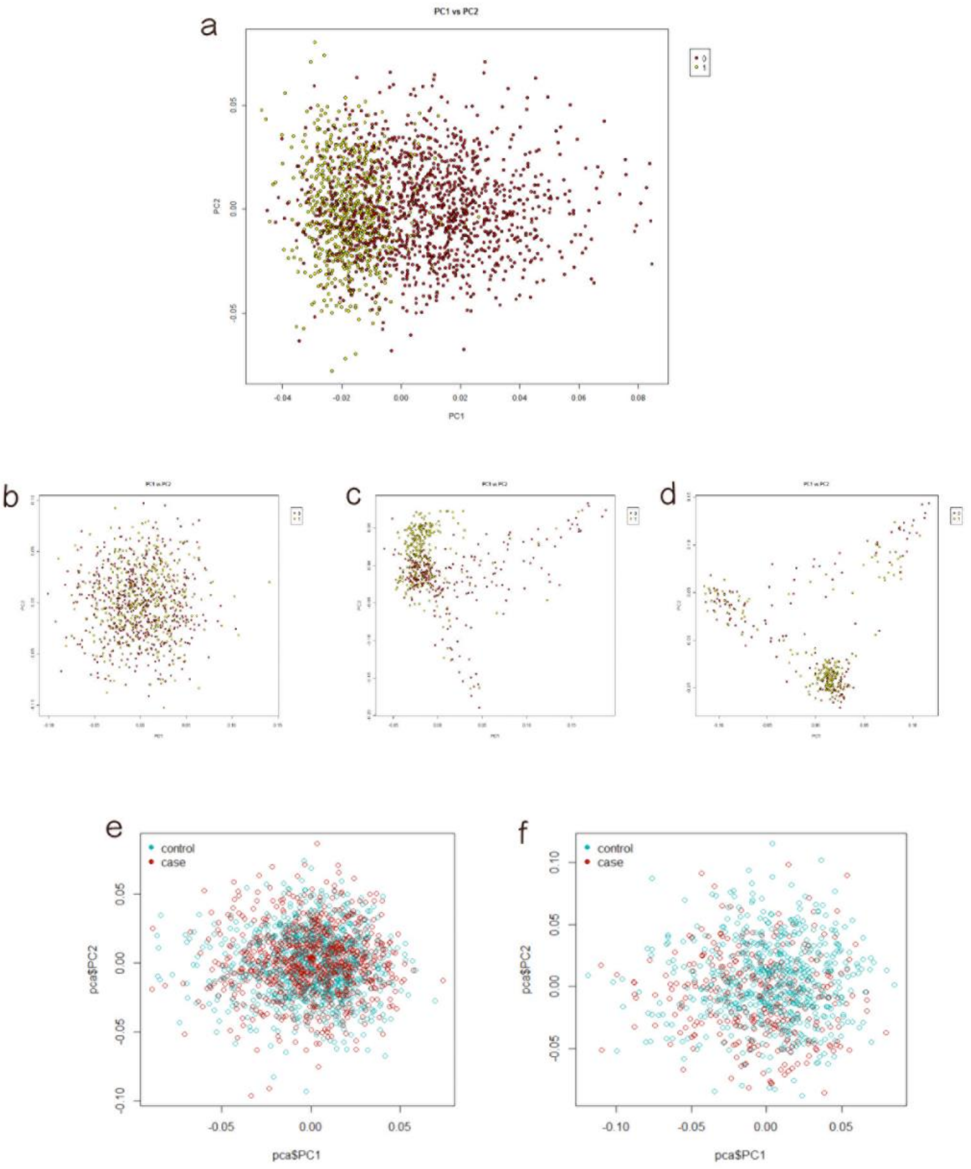

Supplementary Figure 2 Quantile-quantile plot of the associations

Left panel, before removal of SNPs located within known leprosy loci; Right panel, after removal of SNPs located within the known leprosy loci. Dotted vertical line in right panel shows the point where the statistics lift-off from the expected null distribution (between –log10(P) of 2 to 3).

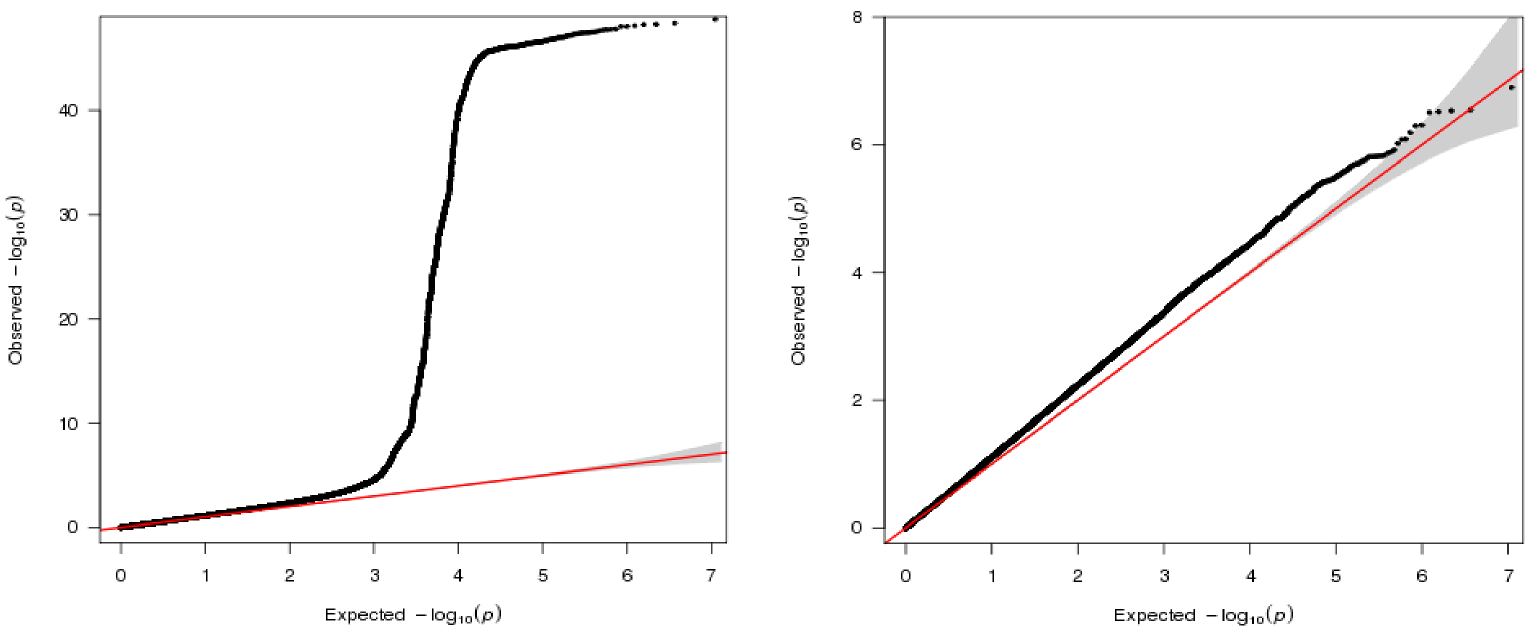

Supplementary Figure 3 Overall genetic architecture of leprosy across functional categories and tissues.

Enrichment estimates for the main annotations and tissues of LDSC. Error bars represent 95% confidence intervals around the estimate. Categories are sorted by P value, with boxes indicating annotations or tissues that pass the multiple testing significance threshold. CNS, central nervous system;; DHS, DNase hypersenstivity; GI, gastrointestinal; TFBS, transcription factor binding site; Tss, transcription start site; UTR, untranslated region.

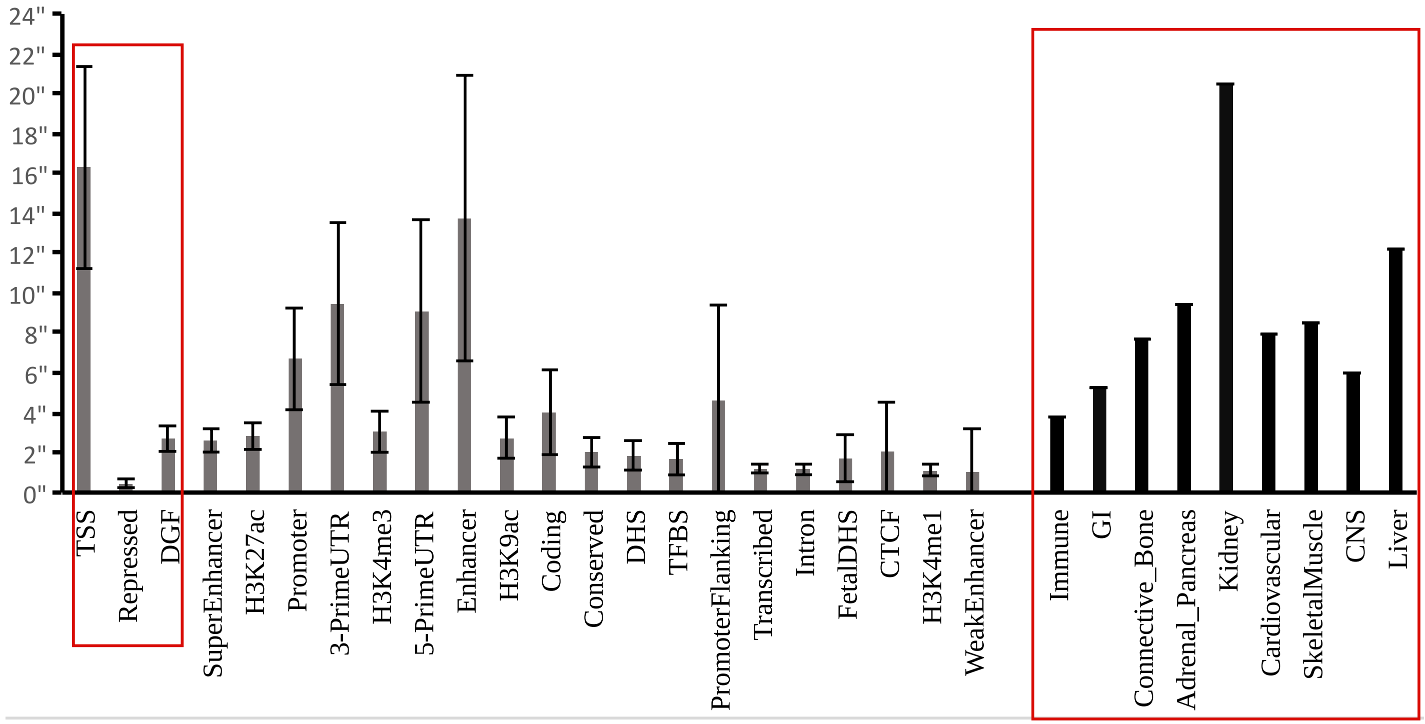

Supplementary Tables

Supplementary Table 1 Baseline characteristics of cases and controls

	CASES									CONTROLS							
					Ethnicity								Ethnicity				
	N	Male/ Female	Mean age	Mean age at onset	North Han	Sichuan Han	Yunnan Han	Guizhou Han	South Minority	N	Male/ Female	Mean age	North Han	Sichuan Han	Yunnan Han	Guizhou Han	South Minority
Discovery Study 1	706	555/151	65.5	21.8	706	-	-	-	-	1223	669/554	34.9	1223	-	-	-	-
Discovery Study 2	840	634/206	60.0	23.2	374	265	-	-	201	924	608/316	43.7	510	263	-	-	151
Discovery Study 3	1197	921/276	64.0	21.1	864	-	333	-	-	1426	975/451	47.6	853	-	573	-	-
Replication Phase 1	1516	1195/321	64.6	23.0	1516	-	-	-	-	1512	1004/508	45.5	1512	-	-	-	-
Replication Phase 2	3897	2929/968	61.2	26.9	1666	906	829	496	-	10525	6453/4072	48.0	8259	878	589	799	-
Total/Mean	8156	6234/1922	62.5	24.5	5126	1171	1162	496	201	15610	9709/5901	46.4	12357	1141	1162	799	151

*The location of North Han, Sichuan, Yunan, Guizhou were showed in supplementary figure 3

Supplementary Table 2 Association results of 127 replicated SNPs in Stage 2

CHR	SNP	BP	A1	A2	F_A	F_U	OR	L95	U95	P
1	rs76381565	36868085	T	C	0.302	0.295	1.037	0.928	1.160	5.18E-01
1	rs11207509	60174951	A	G	0.381	0.377	1.018	0.907	1.144	7.58E-01
1	rs17508449	114085145	T	C	0.147	0.169	0.849	0.739	0.975	2.02E-02
1	rs1217222	114141635	C	T	0.208	0.222	0.921	0.814	1.043	1.95E-01
1	rs1166333	217258449	A	G	0.413	0.441	0.931	0.847	1.023	1.35E-01
1	rs17359468	114242706	A	G	0.123	0.142	0.846	0.720	0.994	4.25E-02
1	rs17459496	116694130	C	T	0.236	0.250	0.929	0.826	1.046	2.26E-01
1	rs7417366	168463587	A	G	0.274	0.305	0.864	0.766	0.974	1.66E-02
1	rs6694610	187860812	G	C	0.110	0.101	1.107	0.937	1.309	2.33E-01
2	rs16866054	7407863	G	T	0.425	0.421	1.016	0.916	1.128	7.58E-01
2	rs74846502	36702265	G	A	0.056	0.065	0.846	0.684	1.047	1.23E-01
2	rs62165062	76451146	G	C	0.385	0.383	1.009	0.899	1.132	8.80E-01
2	rs13417117	104033820	A	G	0.115	0.109	1.057	0.904	1.237	4.88E-01
2	rs7574846	120973491	G	A	0.266	0.268	0.992	0.884	1.113	8.93E-01
2	rs1257205	134980532	A	G	0.096	0.080	1.141	0.710	1.836	5.86E-01
2	rs1349159	138235279	T	C	0.341	0.326	1.073	0.962	1.196	2.05E-01
2	rs3863915	154305485	A	G	0.044	0.048	0.916	0.727	1.155	4.60E-01
2	rs834128	158066572	T	C	0.406	0.427	0.920	0.830	1.019	1.10E-01
2	rs10180790	167293853	C	T	0.467	0.472	0.980	0.882	1.087	6.98E-01
2	rs79037144	188917817	G	A	0.228	0.236	0.957	0.848	1.080	4.77E-01
2	rs1208070	202559964	C	T	0.209	0.196	1.084	0.954	1.231	2.16E-01
2	rs4663905	239456687	G	C	0.074	0.071	1.042	0.862	1.260	6.69E-01
3	rs13066288	4343387	G	A	0.111	0.106	1.055	0.885	1.258	5.49E-01
3	rs6807915	12313846	C	T	0.465	0.501	0.870	0.787	0.961	6.28E-03
3	rs141118348	56502618	T	C	0.395	0.313	1.352	1.183	1.544	8.85E-06
3	rs13079104	81886039	A	T	0.407	0.391	1.071	0.956	1.201	2.37E-01
3	rs6437513	104112018	A	C	0.233	0.247	0.922	0.819	1.038	1.81E-01
3	rs12635287	136759378	G	A	0.316	0.329	0.939	0.842	1.048	2.62E-01
3	rs6793154	151160595	C	T	0.118	0.099	1.216	1.019	1.452	3.01E-02
3	rs79927413	189851926	T	A	0.000	0.006	0.000	0.000	inf	9.97E-01
4	rs393635	2802113	A	G	0.489	0.417	1.368	1.160	1.614	1.96E-04
4	rs10020821	5445587	A	G	0.005	0.002	2.007	0.849	4.749	1.13E-01
4	rs59222941	82848918	C	T	0.103	0.098	1.056	0.892	1.250	5.24E-01
4	rs6534907	133743326	T	C	0.178	0.201	0.864	0.751	0.994	4.04E-02
4	rs77280512	176629971	T	C	0.266	0.235	1.186	1.054	1.336	4.79E-03
4	rs72715458	181224835	A	G	0.118	0.141	0.819	0.689	0.973	2.34E-02
5	rs10941002	32214800	C	A	0.356	0.349	1.031	0.926	1.149	5.75E-01

5	rs11952663	35811721	C	G	0.049	0.068	0.727	0.589	0.897	2.92E-03
5	rs16890226	59109052	G	C	0.129	0.121	1.073	0.916	1.258	3.83E-01
5	rs11742057	73059226	C	G	0.128	0.135	0.944	0.812	1.097	4.50E-01
5	rs146238250	103291974	G	A	0.022	0.032	0.674	0.358	1.270	2.22E-01
5	rs61535452	135058737	T	C	0.445	0.435	1.041	0.938	1.155	4.55E-01
5	rs184798074	137306826	G	C	0.000	0.001	0.000	0.000	inf	9.99E-01
5	rs1428920	152877008	C	T	0.306	0.314	0.962	0.862	1.074	4.95E-01
5	rs544421	172376178	A	G	0.185	0.174	1.076	0.944	1.227	2.71E-01
5	rs730977	180600645	C	T	0.050	0.047	1.076	0.849	1.364	5.46E-01
6	rs117373998	40265390	T	C	0.070	0.070	0.999	0.824	1.212	9.95E-01
6	rs477011	44635740	G	A	0.051	0.053	0.962	0.771	1.201	7.35E-01
6	rs17518759	104846730	T	G	0.146	0.152	0.954	0.827	1.101	5.23E-01
6	rs76996465	144389939	T	C	0.150	0.122	1.267	1.093	1.468	1.72E-03
6	rs76944937	144446830	C	T	0.379	0.170	3.262	2.194	4.850	5.18E-09
6	rs11756359	145639105	C	T	0.050	0.068	0.745	0.604	0.919	5.93E-03
6	rs9455554	168386212	T	C	0.380	0.386	0.973	0.874	1.082	6.09E-01
7	rs2723411	13477607	T	C	0.487	0.464	1.096	0.988	1.215	8.20E-02
7	rs11760340	15776274	T	A	0.195	0.198	0.982	0.863	1.117	7.78E-01
7	rs78656787	17267538	C	T	0.013	0.011	1.124	0.708	1.784	6.20E-01
7	rs4720118	33469241	T	C	0.385	0.337	1.202	1.085	1.331	4.15E-04
7	rs856540	46708387	A	G	0.130	0.115	1.146	0.981	1.338	8.51E-02
7	rs12674212	80866531	G	A	0.193	0.115	1.828	1.066	3.133	2.83E-02
7	rs7777958	93892286	C	A	0.215	0.206	1.054	0.931	1.194	4.03E-01
7	rs2952571	121067486	T	C	0.318	0.326	0.963	0.853	1.087	5.38E-01
7	rs13238442	124340390	G	A	0.095	0.099	0.958	0.807	1.136	6.18E-01
7	rs1008274	126544422	T	C	0.320	0.323	0.987	0.884	1.101	8.08E-01
7	rs56244353	146586044	A	G	0.344	0.324	1.095	0.983	1.219	9.94E-02
8	rs56129402	3527820	C	A	0.429	0.437	0.967	0.873	1.072	5.22E-01
8	rs12675556	4885120	A	T	0.434	0.416	1.078	0.973	1.195	1.53E-01
8	rs55894533	11749242	C	A	0.435	0.394	1.188	1.071	1.318	1.14E-03
8	rs17609949	15037929	C	T	0.487	0.466	1.092	0.983	1.212	1.01E-01
8	rs2681619	31321243	G	T	0.369	0.355	1.063	0.957	1.182	2.53E-01
8	rs3134240	96573370	A	G	0.102	0.099	1.031	0.871	1.220	7.24E-01
8	rs7830756	105268990	C	T	0.159	0.141	1.160	1.004	1.340	4.34E-02
8	rs79473992	108581658	A	G	0.058	0.058	1.011	0.816	1.253	9.21E-01
8	rs10100465	118626279	A	G	0.244	0.273	0.868	0.774	0.973	1.48E-02
8	rs7003629	129017012	G	C	0.236	0.227	1.056	0.936	1.190	3.77E-01
8	rs17731990	134838926	T	C	0.158	0.142	1.135	0.985	1.307	7.97E-02
9	rs301437	4587560	T	C	0.244	0.274	0.856	0.762	0.962	8.83E-03
9	rs6477368	9083516	C	T	0.053	0.070	0.760	0.617	0.937	1.01E-02

9	rs12380481	16791865	T	C	0.193	0.174	1.121	0.990	1.270	7.17E-02
9	rs10972319	35112224	G	A	0.021	0.020	1.087	0.764	1.546	6.43E-01
9	rs78607317	71547087	G	A	0.014	0.008	1.633	0.994	2.682	5.30E-02
9	rs13293006	77136896	A	C	0.158	0.143	1.128	0.977	1.301	9.96E-02
9	rs9722950	79339123	G	A	0.042	0.068	0.698	0.100	4.894	7.18E-01
9	rs1577808	120226415	G	T	0.320	0.312	1.039	0.930	1.162	4.99E-01
10	rs4589237	33765303	T	C	0.018	0.023	0.773	0.536	1.115	1.68E-01
10	rs10900290	43471575	T	C	0.347	0.343	1.014	0.910	1.129	8.03E-01
10	rs7069877	71488757	G	T	0.284	0.277	1.039	0.915	1.180	5.51E-01
10	rs942793	81048158	T	G	0.286	0.313	0.861	0.763	0.972	1.56E-02
10	rs4962415	126693935	T	C	0.020	0.023	0.864	0.603	1.237	4.25E-01
11	rs4758330	8329193	T	C	0.190	0.206	0.903	0.796	1.025	1.14E-01
11	rs11035198	39280831	T	C	0.406	0.412	0.975	0.878	1.082	6.27E-01
11	rs11037957	44354596	A	G	0.378	0.418	0.842	0.759	0.935	1.23E-03
11	rs1117857	82392514	G	C	0.288	0.271	1.094	0.975	1.229	1.27E-01
11	rs10898436	85841148	C	A	0.405	0.385	1.093	0.984	1.214	9.86E-02
11	rs10891071	110045141	G	A	0.387	0.387	1.000	0.900	1.112	9.95E-01
11	rs7943463	121850960	C	T	0.050	0.080	0.611	0.483	0.772	3.60E-05
12	rs6489652	711811	A	G	0.319	0.347	0.884	0.795	0.982	2.14E-02
12	rs7311031	40525592	G	A	0.229	0.255	0.886	0.794	0.989	3.08E-02
12	rs1292012	115815441	A	C	0.360	0.351	1.042	0.936	1.159	4.53E-01
13	rs7998422	24189126	G	A	0.480	0.507	0.896	0.801	1.002	5.40E-02
13	rs17057348	38755621	C	T	0.109	0.110	0.990	0.840	1.167	9.05E-01
13	rs78928712	44186036	T	C	0.093	0.080	1.179	0.969	1.434	1.01E-01
13	rs2812245	51308055	G	A	0.465	0.434	1.136	1.017	1.269	2.37E-02
14	rs72678270	54323380	A	G	0.136	0.147	0.916	0.791	1.060	2.40E-01
14	rs72724236	56936967	C	T	0.197	0.202	0.970	0.855	1.099	6.31E-01
14	rs7143018	61717064	C	T	0.039	0.151	0.345	0.153	0.780	1.05E-02
14	rs6573776	67979352	C	T	0.430	0.433	0.986	0.889	1.095	7.94E-01
14	rs1682540	87740795	G	A	0.115	0.128	0.892	0.768	1.037	1.36E-01
15	rs12594044	47617719	G	A	0.225	0.224	1.003	0.891	1.129	9.61E-01
15	rs9888726	61644080	T	C	0.404	0.415	0.951	0.854	1.058	3.56E-01
15	rs77979355	75070518	A	G	0.241	0.193	1.333	1.176	1.511	7.23E-06
15	rs6496271	98513629	G	T	0.471	0.478	0.971	0.875	1.078	5.81E-01
16	rs34411505	27406689	G	A	0.140	0.159	0.856	0.739	0.991	3.74E-02
17	rs12602313	2303387	T	C	0.272	0.287	0.926	0.826	1.039	1.92E-01
17	rs8067890	39914070	T	G	0.447	0.475	0.894	0.808	0.989	2.98E-02
17	rs4380108	60893485	T	C	0.287	0.292	0.976	0.873	1.092	6.71E-01
17	rs8080960	69786943	G	A	0.118	0.124	0.942	0.805	1.102	4.54E-01
17	rs7503888	72004228	T	C	0.264	0.273	0.957	0.854	1.074	4.58E-01

18	rs1445093	49770823	A	G	0.435	0.428	1.029	0.929	1.140	5.85E-01
18	rs9950773	74021181	A	G	0.362	0.341	1.094	0.983	1.217	9.95E-02
19	rs7251792	1021725	A	C	0.283	0.292	0.959	0.858	1.072	4.59E-01
20	rs243899	13238759	A	G	0.354	0.364	0.956	0.859	1.063	4.06E-01
20	rs58065519	47941646	T	C	0.045	0.045	1.016	0.794	1.300	9.00E-01
20	rs118112362	50381707	T	C	0.028	0.029	0.964	0.711	1.307	8.12E-01
21	rs232418	22817724	C	T	0.181	0.173	1.056	0.926	1.204	4.17E-01
21	rs1888497	41533905	T	C	0.095	0.086	1.113	0.917	1.351	2.78E-01
22	rs2097465	36551916	T	C	0.086	0.091	0.945	0.794	1.125	5.27E-01
22	rs5770312	49767684	A	G	0.125	0.130	0.960	0.825	1.116	5.92E-01

A1 is the minor allele, while F_A represents allele frequency in cases and F_U represents allele frequency in controls.

Supplementary Table 3 Association results of 21 replicated SNPs in Stage 3

SNP info					meta of stage3				meta all			
CHR	BP	SNP	A1	A2	P	OR	Q	I	P	OR	Q	I
1	114085145	rs17508449	T	C	1.56E-02	0.912	0.887	0	4.71E-06	0.878	0.704	0
1	114242706	rs17359468	A	G	3.04E-02	0.915	0.568	0	1.18E-05	0.873	0.396	3.27
1	168463587	rs7417366	A	G	8.22E-03	0.923	0.000	98.05	4.81E-08	0.883	0.000	96.87
**3**	**12313846**	**rs6807915**	**C**	**T**	**2.84E-04**	**0.901**	**0.915**	**0**	**1.94E-08**	**0.887**	**0.947**	**0**
3	151160595	rs6793154	C	T	3.46E-02	1.099	0.136	45.9	2.55E-05	1.155	0.120	42.82
4	176629971	rs77280512	T	C	6.37E-02	1.061	0.539	0	1.74E-05	1.110	0.241	25.76
**4**	**181224835**	**rs72715458**	**A**	**G**	**9.08E-04**	**0.871**	**0.134**	**46.2**	**2.62E-07**	**0.849**	**0.266**	**22.38**
5	35811721	rs11952663	C	G	4.42E-01	0.958	0.712	0	5.49E-05	0.847	0.033	58.81
6	144389939	rs76996465	T	C	6.69E-02	1.082	0.138	45.54	2.07E-06	1.165	0.028	60.13
6	145639105	rs11756359	C	T	1.09E-20	1.821	0.000	97.65	2.64E-01	1.040	0.000	97.89
**7**	**33469241**	**rs4720118**	**T**	**C**	**1.03E-03**	**1.119**	**0.638**	**0**	**3.85E-10**	**1.161**	**0.551**	**0**
**8**	**11749242**	**rs55894533**	**C**	**A**	**2.26E-06**	**1.148**	**0.080**	**55.6**	**5.07E-11**	**1.154**	**0.212**	**29.82**
8	105268990	rs7830756	C	T	3.09E-01	1.040	0.117	49.1	1.41E-04	1.118	0.009	67.25
**8**	**118626279**	**rs10100465**	**A**	**G**	**9.27E-06**	**0.863**	**0.008**	**74.55**	**2.85E-11**	**0.850**	**0.025**	**61.12**
9	4587560	rs301437	T	C	2.15E-01	0.962	0.196	35.99	4.10E-05	0.908	0.031	59.38
10	81048158	rs942793	T	G	1.96E-04	0.891	0.953	0	1.68E-09	0.869	0.831	0
11	44354596	rs11037957	A	G	8.23E-01	0.994	0.145	44.35	3.78E-04	0.926	0.002	72.92
13	51308055	rs2812245	G	A	8.53E-01	1.005	0.718	0	4.24E-02	1.045	0.306	16.72
15	75070518	rs77979355	A	G	2.12E-02	1.088	0.162	41.55	6.70E-10	1.176	0.009	67.19
**16**	**27406689**	**rs34411505**	**G**	**A**	**8.05E-03**	**0.896**	**0.091**	**53.54**	**5.82E-07**	**0.858**	**0.083**	**48.6**

*Rs7417366, rs942793, rs77979355 were not reported in the manuscript due to either failed of HWE test or significant Q value.

Supplementary Table 4 eQTL analysis of four novel associations

Lead SNP	Study ID	Paper_title	Tissue	Correlated-gene	p-value	eQTL SNP	r2 with lead SNP	D' with lead SNP
rs6807915	Westra 2013	Systematic identification of trans eQTLs as putative drivers of known disease associations	Whole_Blood	SYN2	1.74E-04	rs9853146	0.95	0.98
rs6807915	Westra 2013	Systematic identification of trans eQTLs as putative drivers of known disease associations	Whole_Blood	SYN2	7.69E-05	rs7610451	0.92	0.97
rs6807915	Westra 2013	Systematic identification of trans eQTLs as putative drivers of known disease associations	Whole_Blood	SYN2	1.80E-04	rs9823123	0.91	0.96
rs6807915	Westra 2013	Systematic identification of trans eQTLs as putative drivers of known disease associations	Whole_Blood	SYN2	1.83E-04	rs7632481	0.94	0.97
rs4720118	GTEx2015_v6	The Genotype-Tissue Expression (GTEx) pilot analysis: Multitissue gene regulation in humans	Thyroid	BBS9	2.81E-07	rs4720118	1	1
rs4720118	Westra 2013	Systematic identification of trans eQTLs as putative drivers of known disease associations	Whole_Blood	BBS9	3.18E-04	rs4720118	1	1
rs55894533	GTEx2015_v6	The Genotype-Tissue Expression (GTEx) pilot analysis: Multitissue gene regulation in humans	Cells_Transformed_fibroblasts	CTSB	7.48E-18	rs55894533	1	1
rs55894533	GTEx2015_v6	The Genotype-Tissue Expression (GTEx) pilot analysis: Multitissue gene regulation in humans	Whole_Blood	CTSB	1.35E-09	rs55894533	1	1
rs10100465	Westra 2013	Systematic identification of trans eQTLs as putative drivers of known disease associations	Whole_Blood	MED30	4.38E-05	rs10100465	1	1

*Lead SNP represents the current reported associations within the genomic region. eQTL SNP represents those SNPs reported in the publications with listed paper title, which were in high LD (r2>0.9 &D' >0.9) with lead SNPs

Supplementary Table 5 Association results of HLA imputation

Classical HLA allele	P	OR	Q	P_con	OR_con
HLA_A_01	6.37E-08	0.51	0.32	1.49E-07	0.51
HLA_A_0101	6.37E-08	0.51	0.32	1.49E-07	0.51
HLA_A_02	5.74E-01	1.03	0.68	7.67E-01	1.01
HLA_A_0201	7.32E-01	1.02	0.78	8.23E-01	0.99
HLA_A_0203	2.99E-02	1.31	0.66	1.43E-02	1.37
HLA_A_0206	5.60E-02	1.16	0.48	1.81E-01	1.11
HLA_A_0207	1.05E-03	0.77	0.96	9.38E-03	0.81
HLA_A_0211	5.83E-01	0.51	1.00	7.61E-01	0.69
HLA_A_0216	9.99E-01	0.00	1.00	9.99E-01	0.00
HLA_A_03	1.28E-01	0.84	0.40	1.34E-01	0.84
HLA_A_0301	1.28E-01	0.84	0.40	1.34E-01	0.84
HLA_A_0302	9.99E-01	0.00	1.00	9.99E-01	0.00
HLA_A_11	7.94E-04	1.18	0.37	4.70E-03	1.16
HLA_A_1101	4.55E-04	1.20	0.35	2.78E-03	1.17
HLA_A_1102	6.09E-01	0.92	0.92	5.49E-01	0.90
HLA_A_2402	5.10E-01	0.96	0.10	1.10E-01	0.91
HLA_A_2403	8.64E-01	1.42	1.00	9.13E-01	1.29
HLA_A_2407	4.01E-01	0.80	0.94	6.58E-01	0.89
HLA_A_2410	8.60E-01	1.22	1.00	8.41E-01	1.27
HLA_A_26	8.56E-01	1.02	0.65	8.24E-01	0.97
HLA_A_2601	8.56E-01	1.02	0.65	8.24E-01	0.97
HLA_A_2603	1.00E+00	0.00	1.00	1.00E+00	0.01
HLA_A_29	9.05E-02	1.38	0.76	3.90E-02	1.49

HLA_A_2901	9.05E-02	1.38	0.76	3.90E-02	1.49
HLA_A_2902	6.74E-01	0.76	1.00	6.27E-01	0.73
HLA_A_30	5.89E-01	1.04	0.67	2.57E-02	1.19
HLA_A_3001	5.89E-01	1.04	0.67	2.57E-02	1.19
HLA_A_31	3.18E-01	1.11	0.78	9.78E-01	1.00
HLA_A_3101	3.18E-01	1.11	0.78	9.78E-01	1.00
HLA_A_32	4.82E-01	0.89	0.73	9.75E-01	0.99
HLA_A_3201	4.82E-01	0.89	0.73	9.75E-01	0.99
HLA_A_33	2.12E-02	0.85	0.25	2.11E-01	0.91
HLA_A_3303	1.93E-02	0.84	0.25	1.99E-01	0.91
HLA_A_34	7.46E-01	2.18	1.00	8.07E-01	1.97
HLA_A_3401	7.46E-01	2.18	1.00	8.07E-01	1.97
HLA_A_68	1.02E-02	0.57	0.92	2.25E-02	0.60
HLA_A_6801	1.02E-02	0.57	0.92	2.25E-02	0.60
HLA_A_74	1.00E+00	0.00	1.00	1.00E+00	0.01
HLA_A_7401	1.00E+00	0.00	1.00	1.00E+00	0.01
HLA_B_07	4.05E-04	1.42	0.96	1.41E-02	1.28
HLA_B_0702	1.67E-02	1.31	0.82	5.14E-01	1.08
HLA_B_0705	4.55E-03	1.72	0.47	2.84E-04	2.01
HLA_B_08	4.05E-01	0.81	0.73	6.38E-01	0.88
HLA_B_0801	2.82E-01	0.75	0.81	4.61E-01	0.82
HLA_B_13	3.28E-02	1.14	0.21	2.80E-03	1.20
HLA_B_1301	5.70E-05	1.47	0.59	4.97E-03	1.32
HLA_B_1302	6.27E-01	0.96	0.54	1.26E-01	1.12
HLA_B_15	4.71E-05	1.25	0.54	4.45E-02	1.12
HLA_B_1501	2.34E-01	1.09	0.62	9.66E-01	1.00
HLA_B_1502	4.33E-05	1.52	0.80	1.06E-02	1.32

HLA_B_1505	4.36E-01	1.84	0.96	4.45E-01	1.82
HLA_B_1507	7.90E-01	1.31	1.00	6.98E-01	1.49
HLA_B_1508	4.15E-01	1.86	0.96	3.24E-01	2.14
HLA_B_1511	7.69E-01	0.80	1.00	9.09E-01	0.92
HLA_B_1512	7.90E-01	1.31	1.00	6.98E-01	1.49
HLA_B_1513	4.74E-01	0.62	0.83	6.76E-01	0.76
HLA_B_1518	3.38E-04	1.75	0.36	4.39E-03	1.58
HLA_B_1521	6.49E-01	1.37	1.00	5.36E-01	1.53
HLA_B_1525	7.03E-01	0.92	0.10	6.39E-01	0.90
HLA_B_18	8.21E-01	1.07	0.57	4.78E-01	1.24
HLA_B_1801	8.21E-01	1.07	0.57	4.78E-01	1.24
HLA_B_1802	7.90E-01	1.31	1.00	6.98E-01	1.49
HLA_B_2706	2.99E-02	0.56	0.20	2.46E-02	0.54
HLA_B_35	3.52E-01	0.91	0.27	5.92E-02	0.83
HLA_B_3501	9.87E-01	1.00	0.15	1.22E-01	0.83
HLA_B_3503	7.10E-02	0.70	0.62	1.89E-01	0.77
HLA_B_3505	3.99E-01	1.42	0.74	1.88E-01	1.73
HLA_B_3530	7.90E-01	1.31	1.00	6.98E-01	1.49
HLA_B_37	8.01E-03	0.58	0.60	4.58E-02	0.66
HLA_B_3701	8.01E-03	0.58	0.60	4.58E-02	0.66
HLA_B_38	6.63E-01	1.05	0.38	3.11E-01	1.11
HLA_B_3801	7.90E-01	1.31	1.00	6.98E-01	1.49
HLA_B_3802	6.03E-01	1.06	0.37	2.78E-01	1.12
HLA_B_39	3.40E-01	1.20	0.30	2.26E-01	1.27
HLA_B_3901	3.40E-01	1.20	0.30	2.26E-01	1.27
HLA_B_40	1.23E-01	1.09	0.35	4.10E-01	1.05
HLA_B_4001	3.42E-01	1.07	0.12	6.74E-01	1.03

HLA_B_4002	2.97E-01	0.84	0.37	2.73E-01	0.83
HLA_B_4006	4.53E-02	1.19	0.16	1.17E-01	1.15
HLA_B_44	9.20E-02	0.82	0.43	6.38E-01	0.95
HLA_B_4403	9.20E-02	0.82	0.43	6.38E-01	0.95
HLA_B_46	2.83E-03	0.81	0.87	6.74E-02	0.88
HLA_B_4601	2.83E-03	0.81	0.87	6.74E-02	0.88
HLA_B_49	8.28E-01	1.20	1.00	6.73E-01	1.42
HLA_B_4901	8.28E-01	1.20	1.00	6.73E-01	1.42
HLA_B_50	1.34E-01	0.66	0.80	2.34E-01	0.72
HLA_B_5001	1.34E-01	0.66	0.80	2.34E-01	0.72
HLA_B_51	4.54E-01	0.94	0.31	5.04E-01	0.95
HLA_B_5101	4.28E-01	0.94	0.28	4.87E-01	0.94
HLA_B_5102	7.22E-01	1.21	0.99	6.21E-01	1.32
HLA_B_5107	8.28E-01	1.20	1.00	6.73E-01	1.42
HLA_B_52	4.76E-01	0.93	0.77	7.40E-01	1.04
HLA_B_5201	4.76E-01	0.93	0.77	7.40E-01	1.04
HLA_B_54	1.50E-02	0.76	0.97	2.83E-02	0.78
HLA_B_5401	1.50E-02	0.76	0.97	2.83E-02	0.78
HLA_B_55	2.05E-01	1.24	0.95	1.07E-01	1.32
HLA_B_5501	9.05E-01	0.91	1.00	8.95E-01	1.11
HLA_B_5502	2.05E-01	1.24	0.95	1.07E-01	1.32
HLA_B_56	8.62E-02	0.70	0.61	1.41E-01	0.73
HLA_B_5601	1.36E-01	0.74	0.30	2.15E-01	0.77
HLA_B_57	2.97E-03	0.52	0.91	1.46E-02	0.58
HLA_B_5701	2.97E-03	0.52	0.91	1.46E-02	0.58
HLA_B_59	9.05E-01	0.91	1.00	8.95E-01	1.11
HLA_B_5901	9.05E-01	0.91	1.00	8.95E-01	1.11

HLA_B_67	2.20E-02	1.54	0.35	3.74E-02	1.50
HLA_B_6701	2.20E-02	1.54	0.35	3.74E-02	1.50
HLA_C_01	2.73E-05	0.78	0.93	1.64E-03	0.83
HLA_C_0102	2.83E-05	0.78	0.93	1.68E-03	0.83
HLA_C_0103	5.25E-01	1.47	0.99	4.54E-01	1.58
HLA_C_02	9.98E-01	0.00	1.00	9.98E-01	0.00
HLA_C_0202	9.98E-01	0.00	1.00	9.98E-01	0.00
HLA_C_03	2.13E-02	1.12	0.30	7.83E-01	1.01
HLA_C_0303	2.28E-02	1.19	0.41	9.87E-01	1.00
HLA_C_0304	2.40E-03	1.23	0.17	1.15E-01	1.12
HLA_C_0403	4.44E-01	0.85	0.26	5.25E-01	0.88
HLA_C_0406	9.99E-01	0.00	1.00	9.99E-01	0.00
HLA_C_06	1.50E-03	0.81	0.31	3.40E-01	0.94
HLA_C_0602	1.76E-03	0.82	0.39	3.60E-01	0.94
HLA_C_07	1.72E-01	1.08	0.57	2.10E-01	1.07
HLA_C_0701	1.03E-01	0.77	0.84	4.34E-01	0.88
HLA_C_0702	1.79E-01	1.08	0.60	3.29E-01	1.06
HLA_C_0704	5.90E-02	1.40	0.83	8.11E-02	1.38
HLA_C_0726	6.17E-01	2.05	1.00	5.72E-01	2.28
HLA_C_12	6.57E-01	0.96	0.19	4.60E-01	1.07
HLA_C_1202	6.89E-01	0.96	0.43	6.45E-01	1.05
HLA_C_1203	7.50E-01	0.96	0.28	6.22E-01	1.07
HLA_C_1204	6.17E-01	2.05	1.00	5.72E-01	2.28
HLA_C_14	1.38E-01	0.86	0.39	1.95E-01	0.88
HLA_C_1402	4.32E-01	0.92	0.55	4.10E-01	0.92
HLA_C_1403	1.53E-02	0.51	0.91	6.59E-02	0.60
HLA_C_15	9.02E-01	1.01	0.46	6.15E-01	0.95

HLA_C_1502	5.55E-01	0.94	0.07	1.22E-01	0.84
HLA_C_1505	2.66E-01	1.31	0.80	1.28E-01	1.46
HLA_C_1507	6.17E-01	2.05	1.00	5.72E-01	2.28
HLA_C_16	8.20E-01	1.10	0.97	6.42E-01	1.22
HLA_C_1602	8.20E-01	1.10	0.97	6.42E-01	1.22
HLA_DPA1_01	7.11E-02	1.08	0.78	7.87E-02	1.10
HLA_DPA1_0103	6.85E-02	1.08	0.78	7.39E-02	1.10
HLA_DPA1_0104	3.25E-01	3.15	1.00	1.00E+00	8.14
HLA_DPA1_02	1.34E-01	1.06	0.25	1.10E-01	1.09
HLA_DPA1_0201	8.83E-02	0.90	0.32	9.20E-01	1.01
HLA_DPA1_0202	4.35E-01	0.97	0.76	2.17E-02	0.87
HLA_DPA1_04	5.08E-01	0.91	0.22	9.20E-01	0.99
HLA_DPA1_0401	5.08E-01	0.91	0.22	9.20E-01	0.99
HLA_DPB1_01	8.01E-01	1.23	1.00	7.12E-01	1.69
HLA_DPB1_0101	8.01E-01	1.23	1.00	7.12E-01	1.69
HLA_DPB1_02	7.72E-02	1.08	0.73	6.46E-01	0.97
HLA_DPB1_0201	8.76E-03	1.13	0.60	6.85E-01	1.03
HLA_DPB1_0202	2.89E-01	0.92	0.68	1.73E-01	0.87
HLA_DPB1_03	2.23E-02	1.25	0.52	5.54E-02	1.26
HLA_DPB1_0301	2.07E-02	1.26	0.51	5.15E-02	1.26
HLA_DPB1_04	9.79E-01	1.00	0.44	1.16E-01	1.12
HLA_DPB1_0401	7.25E-02	0.88	0.75	5.61E-01	0.95
HLA_DPB1_0402	2.97E-02	1.19	0.34	2.25E-03	1.38
HLA_DPB1_05	9.01E-02	0.93	0.84	8.56E-02	0.91
HLA_DPB1_0501	9.01E-02	0.93	0.84	8.56E-02	0.91
HLA_DPB1_09	9.24E-02	0.79	0.68	4.18E-01	0.86
HLA_DPB1_0901	9.24E-02	0.79	0.68	4.18E-01	0.86

HLA_DPB1_100	1.00E+00	0.79	1.00	1.00E+00	0.98
HLA_DPB1_13	2.38E-01	0.90	0.94	6.62E-01	0.95
HLA_DPB1_1301	2.38E-01	0.90	0.94	6.62E-01	0.95
HLA_DPB1_14	9.75E-01	1.00	0.46	9.56E-01	0.99
HLA_DPB1_1401	9.75E-01	1.00	0.46	9.56E-01	0.99
HLA_DPB1_16	3.33E-01	3.10	1.00	#N/A	#N/A
HLA_DPB1_1601	3.33E-01	3.10	1.00	#N/A	#N/A
HLA_DPB1_17	3.97E-01	0.93	0.85	4.86E-01	1.09
HLA_DPB1_1701	4.47E-01	0.93	0.85	4.44E-01	1.10
HLA_DPB1_19	9.84E-01	1.00	0.58	9.86E-01	1.01
HLA_DPB1_1901	8.98E-01	1.03	0.54	9.11E-01	1.03
HLA_DPB1_21	4.60E-01	1.13	0.40	7.04E-01	1.08
HLA_DPB1_2101	4.60E-01	1.13	0.40	7.04E-01	1.08
HLA_DPB1_26	1.04E-01	1.81	0.96	6.97E-02	2.29
HLA_DPB1_2601	1.04E-01	1.81	0.96	6.97E-02	2.29
HLA_DPB1_28	2.85E-01	0.72	0.12	4.61E-01	0.79
HLA_DPB1_2801	2.85E-01	0.72	0.12	4.61E-01	0.79
HLA_DQA1_01	1.73E-28	1.60	0.25	4.84E-07	1.35
HLA_DQA1_0101	6.47E-01	1.03	0.69	2.73E-03	1.26
HLA_DQA1_0103	5.05E-01	1.05	0.63	3.05E-02	1.21
HLA_DQA1_02	6.70E-04	0.80	0.64	1.73E-01	0.88
HLA_DQA1_0201	6.70E-04	0.80	0.64	1.73E-01	0.88
HLA_DQA1_03	4.26E-11	0.72	0.12	7.39E-03	0.84
HLA_DQA1_0301	3.92E-11	0.72	0.12	7.39E-03	0.84
HLA_DQA1_04	8.87E-02	1.50	0.56	9.96E-03	2.05
HLA_DQA1_0401	8.87E-02	1.50	0.56	9.96E-03	2.05
HLA_DQA1_05	3.62E-09	0.70	0.43	8.68E-05	0.75

HLA_DQA1_0501	3.62E-09	0.70	0.43	8.68E-05	0.75
HLA_DQB1_02	6.20E-07	0.74	0.25	1.17E-03	0.77
HLA_DQB1_0201	6.20E-07	0.74	0.25	1.17E-03	0.77
HLA_DQB1_03	2.25E-09	0.78	0.65	2.73E-01	0.94
HLA_DQB1_0301	6.09E-04	0.84	0.58	2.83E-01	0.93
HLA_DQB1_0302	7.04E-01	1.03	0.27	1.08E-01	1.20
HLA_DQB1_0303	1.57E-06	0.75	0.15	2.02E-01	0.91
HLA_DQB1_04	1.12E-07	0.55	0.10	1.19E-03	0.64
HLA_DQB1_0401	2.10E-11	0.38	0.33	1.43E-06	0.42
HLA_DQB1_0402	5.83E-01	1.11	0.45	1.76E-01	1.35
HLA_DQB1_05	1.08E-01	1.09	0.48	9.35E-04	1.25
HLA_DQB1_0501	4.14E-02	1.21	0.54	2.94E-03	1.40
HLA_DQB1_0502	3.73E-01	1.07	0.31	1.55E-01	1.14
HLA_DQB1_0503	6.32E-01	0.95	0.77	3.68E-01	1.12
HLA_DQB1_06	2.38E-28	1.65	0.06	7.02E-03	1.21
HLA_DQB1_0601	2.01E-06	1.37	0.11	4.19E-03	1.28
HLA_DQB1_0602	2.55E-30	2.07	0.18	9.35E-01	1.01
HLA_DQB1_0609	4.73E-02	1.36	0.19	2.36E-01	1.25
HLA_DRB1_01	5.74E-02	1.28	0.70	5.13E-02	1.38
HLA_DRB1_0101	5.74E-02	1.28	0.70	5.13E-02	1.38
HLA_DRB1_03	9.31E-07	0.58	0.14	6.90E-05	0.57
HLA_DRB1_0301	9.31E-07	0.58	0.14	6.90E-05	0.57
HLA_DRB1_0401	1.33E-01	0.77	0.42	6.33E-01	0.90
HLA_DRB1_0403	9.17E-02	0.77	0.51	4.41E-01	0.86
HLA_DRB1_0404	3.75E-01	1.31	0.58	9.15E-01	0.96
HLA_DRB1_0405	2.26E-12	0.40	0.37	5.19E-07	0.44
HLA_DRB1_0406	8.68E-01	1.02	0.09	2.55E-01	1.19

HLA_DRB1_0410	9.99E-01	0.00	1.00	9.99E-01	0.00
HLA_DRB1_07	6.23E-04	0.80	0.70	1.67E-01	0.88
HLA_DRB1_0701	6.23E-04	0.80	0.70	1.67E-01	0.88
HLA_DRB1_08	1.94E-01	1.11	0.50	4.59E-02	1.22
HLA_DRB1_0801	9.63E-01	1.05	1.00	6.55E-01	1.88
HLA_DRB1_0802	3.02E-01	1.28	0.63	6.99E-02	1.66
HLA_DRB1_0803	4.33E-01	1.07	0.57	2.14E-01	1.14
HLA_DRB1_0809	4.77E-01	0.54	1.00	9.99E-01	0.00
HLA_DRB1_09	3.09E-05	0.77	0.25	3.75E-01	0.93
HLA_DRB1_0901	3.09E-05	0.77	0.25	3.75E-01	0.93
HLA_DRB1_10	5.28E-01	0.90	0.56	9.86E-01	1.00
HLA_DRB1_1001	5.28E-01	0.90	0.56	9.86E-01	1.00
HLA_DRB1_11	7.05E-02	0.86	0.65	7.40E-01	0.97
HLA_DRB1_1101	7.05E-02	0.86	0.65	7.40E-01	0.97
HLA_DRB1_1105	9.99E-01	0.00	1.00	9.99E-01	0.00
HLA_DRB1_12	3.25E-04	0.79	0.12	1.95E-01	0.90
HLA_DRB1_1201	9.39E-07	0.52	0.91	1.47E-03	0.58
HLA_DRB1_1203	9.24E-01	1.11	1.00	8.56E-01	0.75
HLA_DRB1_1301	2.88E-02	0.71	0.08	1.56E-01	0.75
HLA_DRB1_1302	9.84E-01	1.00	0.26	2.39E-01	1.18
HLA_DRB1_1312	1.07E-01	0.38	1.00	1.74E-01	0.44
HLA_DRB1_14	3.02E-01	0.92	0.72	1.88E-01	1.13
HLA_DRB1_1401	3.69E-01	0.89	0.86	8.76E-01	1.02
HLA_DRB1_1403	6.15E-01	0.49	1.00	2.66E-01	0.21
HLA_DRB1_1404	5.70E-01	0.93	0.79	2.92E-01	1.16
HLA_DRB1_1405	2.22E-01	0.78	0.65	7.91E-01	1.07
HLA_DRB1_1407	6.15E-01	0.49	1.00	2.66E-01	0.21

HLA_DRB1_15	2.91E-43	2.03	0.21	2.37E-04	1.63
HLA_DRB1_1501	4.21E-44	2.17	0.22	1.00E+00	1.00
HLA_DRB1_1502	1.45E-01	1.17	0.06	8.49E-04	1.55
HLA_DRB1_1504	8.27E-01	0.77	1.00	5.10E-01	0.47
HLA_DRB1_16	1.67E-01	1.18	0.37	6.94E-02	1.28
HLA_DRB1_1602	1.67E-01	1.18	0.37	6.94E-02	1.28

P_con represents the P value after conditioning on HLA-DRB1*15:01

OR_con represents the odds ratio after conditioning on HLA-DRB1*15:01

Supplementary Table 6 Heritability estimates for genome-wide SNPs in leprosy on assumed disease risk.

Category	All SNPs		known region (LD）explained ratio
	Heritability	SE	
liability scale h2 (prevalence = 0.0001)	0.199	0.010	13.53%
